# Design of Meter-Scale Antenna and Signal Detection System for Underground Magnetic Resonance Sounding in Mines

**DOI:** 10.3390/s18030848

**Published:** 2018-03-13

**Authors:** Xiaofeng Yi, Jian Zhang, Tiehu Fan, Baofeng Tian, Chuandong Jiang

**Affiliations:** 1 Key Laboratory of Geophysical Exploration Equipment, Ministry of Education, Jilin University, Changchun 130061, China; yixiaofeng@jlu.edu.cn (X.Y.); FTH@jlu.edu.cn (T.F.); 2 College of Instrumentation and Electrical Engineering, Jilin University, Changchun 130061, China; jianzhang13@mails.jlu.edu.cn

**Keywords:** magnetic resonance sounding, underground, mine, antenna, antenna matching, data process

## Abstract

Magnetic resonance sounding (MRS) is a novel geophysical method to detect groundwater directly. By applying this method to underground projects in mines and tunnels, warning information can be provided on water bodies that are hidden in front prior to excavation and thus reduce the risk of casualties and accidents. However, unlike its application to ground surfaces, the application of MRS to underground environments is constrained by the narrow space, quite weak MRS signal, and complex electromagnetic interferences with high intensities in mines. Focusing on the special requirements of underground MRS (UMRS) detection, this study proposes the use of an antenna with different turn numbers, which employs a separated transmitter and receiver. We designed a stationary coil with stable performance parameters and with a side length of 2 m, a matching circuit based on a Q-switch and a multi-stage broad/narrowband mixed filter that can cancel out most electromagnetic noise. In addition, noises in the pass-band are further eliminated by adopting statistical criteria and harmonic modeling and stacking, all of which together allow weak UMRS signals to be reliably detected. Finally, we conducted a field case study of the UMRS measurement in the Wujiagou Mine in Shanxi Province, China, with known water bodies. Our results show that the method proposed in this study can be used to obtain UMRS signals in narrow mine environments, and the inverted hydrological information generally agrees with the actual situation. Thus, we conclude that the UMRS method proposed in this study can be used for predicting hazardous water bodies at a distance of 7–9 m in front of the wall for underground mining projects.

## 1. Introduction

Magnetic resonance sounding (MRS) is a recently developed geophysical method for detecting groundwater, and it can be applied for investigating and assessing the quantity of groundwater [1] as well as for advanced exploration and early warning of hazardous water bodies for underground construction in mines and tunnels [2]. The MRS detection method applied to an underground environment is also called underground magnetic resonance sounding (UMRS). However, the problems of narrow spaces in mines and tunnels, complex environments, and electromagnetic interference severely limit the applicability of UMRS. Greben et al. [3] used Surface Nuclear Magnetic Resonance (SNMR) MIDI equipment [4] to conduct UMRS-based advanced exploration of deep mines and tunnels in South Africa, Germany, and Canada with a vertically arranged antenna; because of the low amplitudes of acquired signals and strong interference from the surrounding environment, they could not obtain reliable MRS signals. Later, Lin et al. [2] reviewed and discussed the difficulties in applying the MRS method to underground projects and associated key techniques that awaited breakthroughs. For the first time, their subsequent study also succeeded in obtaining the UMRS signals of a water body 2–12 m using a 6 m × 8 m antenna in front of the Changsongling tunnel in Jilin Province of China [5]. In addition, by using a multi-turn-small-coil antenna, Qin et al. [6] conducted a systematic numerical modeling study of UMRS detection using the Shandong University physical test system for tunnel geological prediction, and the obtained results were in good agreement with the known water body structure. However, given that mines have more complex environments than tunnels with, e.g., a higher amount of industrial construction, more complex noise interference, and narrower spaces, there have not yet been any successful cases of applying UMRS to mines.

Currently, there are four coil-layout modules for surface MRS, including (1) an overlapping coil layout for a coincident or separate transmitter and receiver with a side length of 100 m–150 m [1,7]; (2) an array coil layout [8] composed of a rectangular transmitting coil (75 m × 25 m) and multiple smaller square receiving coils (25 m × 25 m); (3) a central coil layout [9] for which an extra 25-m receiving coil is placed at the center of the transmitting coil with a side length of 100 m to improve the signal-to-noise ratio (SNR) in receiving signals; and (4) a multi-turn-small-coil [10] for which a common coil with a total length of 400 m is wound for four turns with a side length of 12.5 m and whose MRS signal quality is better than that of a 6.25-m eight-turn coil. However, these four layouts of coils for surface MRS are too large to use for UMRS detection in mines that usually have quite narrow roadways (square spaces with side lengths of 1.5 m–3 m). To solve this problem, Yi et al. [11] designed a coil scheme in which different turn numbers are used for the antenna to separate the transmitter and receiver (6 m in side length) and successfully applied the scheme to advanced exploration of hazardous water bodies in tunnels [12]. Furthermore, Lin et al. [13] designed a square multi-turn-small-coil antenna (one turn for the transmitter and 80 turns for the receiver) with a side length of 1 m, whose receiving sensitivity could be improved by liquid nitrogen cooling; however, according to their study, the antenna can be used to detect water bodies only within 1.5 m.

The environment of mines is quite complicated; in addition to narrow spaces, there is also severe electromagnetic interference. Although power-line harmonic noises and spiky noises above the ground surface have significantly decayed when reaching the deep subsurface, there are a number of power transmission cables and transformers and many pieces of engineering equipment, which can produce a large quantity of power-line harmonic noises as well as random noises, both of which can severely compromise the acquisition of UMRS signals. To suppress these noises, the front end of the signal receiving circuit usually adopts a parallel resonant model to improve the Q value, but this procedure can result in severe ringing artifacts. On the other hand, although the Q-toggle circuit proposed by Li et al. [14] can reduce the ringing effect, its applicability is quite poor.

Lin et al. [15] proposed using three parallel amplifiers to reduce the preamplifier input noise, and this method can reduce the noise level to below 1 nV$/\sqrt{\mathrm{Hz}}$. In addition to hardware noise-suppression techniques, many previous scholars have provided various software noise-cancelling methods. For instance, Laser et al. [16] proposed that the power-line modeling method could effectively reduce power-line harmonic noises; based on the principle of nonlinear energy, Wan et al. [17] investigated the possibility of detecting small sharp peaks of received signals for MRS signal detection; and Jinag et al. [18] suggested that statistical stacking methods could be adopted to reduce random noises. However, for the environment of mines, noise interferences are quite strong and complicated, and reliable detection of UMRS signals thus requires further improved hardware receiving, acquiring circuits and optimized data reduction schemes.

Regarding the special environment of mines, we designed a stationary 2-m multi-turn antenna with an optimized number of turns each for the transmitting coil and the separate receiving coil. We also developed a matching circuit and a Q-switch circuit to realize a multi-stage broad/narrow band-mixed circuit design for the stationary antenna, based on which we also proposed a software scheme for data reduction. Finally, we applied our newly developed UMRS equipment to conduct advanced exploration of the roadway in Wujiagou Mine, Shandong Province, China, and obtained reliable MRS signals. Based on the extracted MRS signals, we inverted and discussed the distribution of water content and relaxation times of a water body at a distance of 7–9 m inside the roadway sidewall.

## 2. UMRS Method

### 2.1. Basic of UMRS

**Figure 1:**
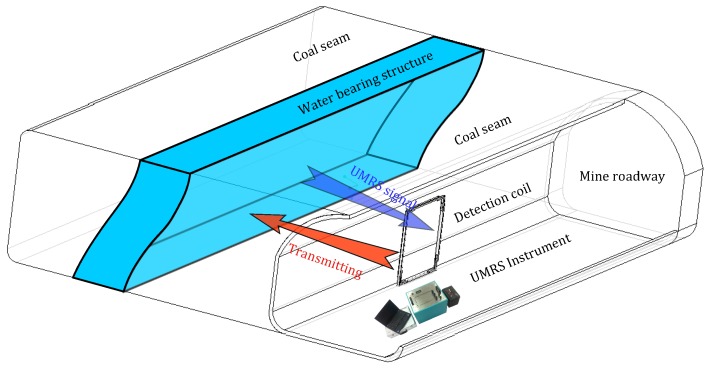
Principle diagram of underground magnetic resonance sounding (UMRS) detection method.

For UMRS detection in mines, the antenna is usually arranged vertically to conduct advanced exploration of a target water body in front or in surrounding rocks, as illustrated in Figure 1. Constrained by the narrow space of the roadway, antennae can be only 2–3 m in size with small effective surface areas. Thus, increasing the number of coil turns is usually adopted to improve the performance of an antenna in detecting weak magnetic fields. Regarding the process of detecting front water bodies, the UMRS method is composed of separated transmitting and receiving processes.

UMRS use the nuclear spins of hydrogen protons in water. Under the geomagnetic field $B_{0}$, the proton spin precess about the axis of $B_{0}$ at the characteristic (angular) Larmor frequency $\omega_{L}=\left|\gamma B_{0}\right|$, where γ is the proton gyromagnetic ratio. In addition to inducing precession, an ensemble of hydrogen protons exhibits a small macroscopic net magnetization $M_{0}$ in thermal equilibrium that is aligned in the direction of $B_{0}$ [19]. The UMRS equipment emits an alternating current (AC) pulse $I\left(t\right)=I_{0}e^{-j\omega_{L}t}$ at the Larmor frequency to excite the proton spins in water, where $I_{0}$ denotes the amplitude and *t* is the time. The transmitting energy can be expressed using a pulse moment $q=I_{0}\times \tau$, where τ denotes the duration of the AC pulse. When hydrogen protons become excited, the direction of the spin magnetization is rotated by some tilting angle, i.e., out of the thermal equilibrium. After switching of the pulse, the spin magnetization relaxes back to the equilibrium, which induces the voltage in the receiving coil, corresponding to a UMRS signal. The amplitude and relaxation time of UMRS signals extracted from acquired data can be used to quantitatively determine the location of a target water body and associated hydrological information, e.g., the water content and the pore structure.

### 2.2. UMRS Signals

The amplitude of a UMRS signal is determined by the size and shape of the chosen antenna as well as the location, thickness, and water content of the water-bearing structure, which can be expressed by a forward-derivation equation based on a theoretical MRS model [20] as follows:

(1) $$V\left(q,t\right)=\int K\left(q,x\right)\cdot w\left(x\right)\cdot e^{-t/T_{2}^{*}\left(x\right)}dx,$$

in which $V\left(q,t\right)$ denotes the induced voltage with an pulse moment of *q* that is received at time *t*; $K\left(q,x\right)$ is the sensitivity of the UMRS signal at a distance of *x* in front of mine wall, called the kernel function; and the water content and the relaxation time of the water body are described by $w\left(x\right)$ (volume percentage, 0 $<w\left(x\right)$ < 100%) and $T_{2}^{*}\left(x\right)$, respectively.

The detailed description of the kernel function was previously given by [21] as follows:

(2) $$\begin{array}{cc} K\left(q,r\right)= & -2\omega_{L}M_{0}\cdot \sin\left(\gamma q\left|{}^{T}B_{⊥}^{+}\left(r\right)\right|\right)\\ & \times \left|{}^{R}B_{⊥}^{-}\left(r\right)\right|\cdot e^{i\cdot \left[\xi_{T}\left(r\right)+\xi_{R}\left(r\right)\right]}\\ & \times \left[{}^{R}{\hat{b}}_{⊥}\left(r\right)\cdot^{T}{\hat{b}}_{⊥}\left(r\right)+i\cdot^{0}\hat{b}\cdot^{R}{\hat{b}}_{⊥}\left(r\right)\times^{T}{\hat{b}}_{⊥}\left(r\right)\right], \end{array}$$

in which r denotes the underground spatial location. $\left|{}^{T}B_{⊥}^{+}\left(r\right)\right|$ is the co-rotating subcomponent of the transmitting magnetic field ${}^{T}B$ after elliptical polarization that is perpendicular to $B_{0}$. $\left|{}^{R}B_{⊥}^{-}\left(r\right)\right|$ is the counter-rotating subcomponent of the transmitting magnetic field ${}^{R}B$ after elliptical polarization that is perpendicular to $B_{0}$. The associated exponents of $\xi_{T}\left(r\right)$ and $\xi_{R}\left(r\right)$ correspond to the phase changes of ${}^{T}B$ and ${}^{R}B$, respectively, after elliptical polarization and ${}^{0}\hat{b}$, ${}^{T}{\hat{b}}_{⊥}\left(r\right)$, and ${}^{R}{\hat{b}}_{⊥}\left(r\right)$ describe the unit vectors $B_{0}$, ${}^{T}B$, and ${}^{R}B$, respectively. For a one-dimensional $K\left(q,x\right)$ function, one need only integrate $K\left(q,r\right)$ along the *y*- and *z*-axes. More details about the forward modelling and the numeral calculation of kernel function can be found in [22].

**Figure 2:**
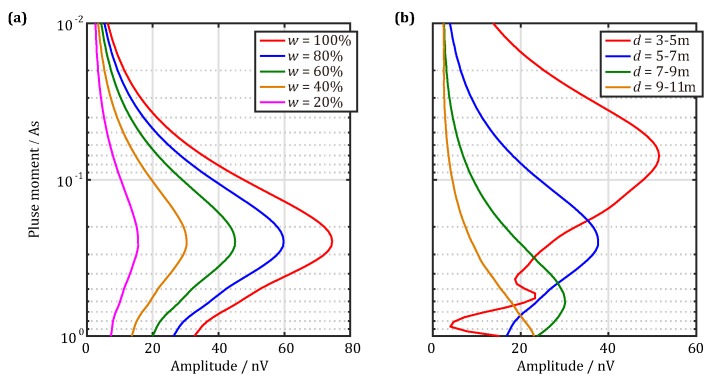
Sounding curves of forward-calculated UMRS signal versus the pulse moment received by the meter-scale antenna for a a 2-m-thick water-bearing layer. (**a**) The center location is 6 m in front and the water content is reduced from 100% to 20%; (**b**) the water content remains 50% and the center location varies from 4 m–10 m.

According to Equations (1) and (2), we obtained the sounding curves of the UMRS signal, the initial amplitudes at t=0 s as functions of pulse moments, as shown in Figure 2. The results in Figure 2a are for a 2-m-thick water-bearing layer 7 m in front and for an antenna consisting of 2-m square antenna transmitting and receiving coils with turn numbers of 18 and 90, respectively. Figure 2a illustrates that, when the water content is reduced from 100% to 20%, the UMRS signal amplitude proportionally decreases from its maximum, 74.5 nV, to 14.9 nV . In addition, Figure 2b represents the sounding curves of the UMRS signal, when the water content remains the same (50%), but the location of the water-bearing layer varies. We can see from Figure 2b that, when the distance between the water-bearing layer and the antenna gradually increases, the UMRS signal amplitude continuously decreases, while the required pulse moment continuously increases. For instance, detecting a 2-m-thick water-bearing layer 4 m in front requires a 0.01-As pulse moment; in comparison, detecting the same thick water-bearing layer 10 m in front requires at least a 1-As pulse moment. Thus, according to the forward-calculated UMRS signals, we conclude that the UMRS signals obtained by a 2-m antenna are quite weak, on the order of only tens of nV, and decrease with increased distance and decreased water content in the water-bearing layer. Thus, for UMRS signal detection, the adopted equipment must exhibit extremely high detection sensitivities.

### 2.3. Inversion Problem

The complex QT inversion scheme is the most advanced algorithm for inverting UMRS signals [23]. The QT inversion constructs a data space $d_{q,t}$ based on all pulse moments *q* and time sequences *t*. This method adopts the gate-integrated method to reduce the data quantity and to improve the SNRs of later data [24] and adds weights to the measured data according to the data errors [25]. It further divides $d_{q,t}$ into a real part and an imaginary part, which together form a data vector d. The modeling space is formed by the water content *w* of each relaxation time $T_{2}^{*}$ and the distance *x* of water-bearing layer and written as m. To obtain a smooth and stable modeling space, the water content between adjacent layer and $T_{2}^{*}$ are processed using a first-order smoothness matrix C, and the regularization parameter λ is continuously adjusted until reaching a balance between the data consistency and the model smoothness. The kernel function is given in Equation (2), which is also divided into a real part and an imaginary part. Thus, the target function for the inversion can be expressed as follows:

(3) $$\Phi =∥d-d^{\mathrm{calc}}{∥}_{2}^{2}+\lambda {∥\mathrm{Cm}∥}_{2}^{2},$$

in which $d^{\mathrm{calc}}$ corresponds to the forward-modelled data calculated by Equation (1). Because of the effects of equipment and data processing, the phase of the forward-calculated data $d^{\mathrm{calc}}$ does not match the phase of the corresponding observational data d, and, in turn, there is a constant phase difference. Thus, before conducting iteration calculations for the inversion, one must first provide a preliminary estimate of the phase difference [23]. The inversion scheme includes two levels of iterative processes. The inner iteration is for a fixed λ and obtains the most optimized result for the current modeling space by conducting a certain number of iterative calculations (e.g., 20 times). In comparison, the external iteration is for multiple λ values, calculates an L-curve for 20 logarithmic sampling points within [10${}^{3}$, 10${}^{6}$], and then determines the most optimized λ according to the turning point of the L-curve [26]. Finally, the uncertainty in the modeling result can be assessed by a recently proposed directed algorithm [27].

**Figure 3:**
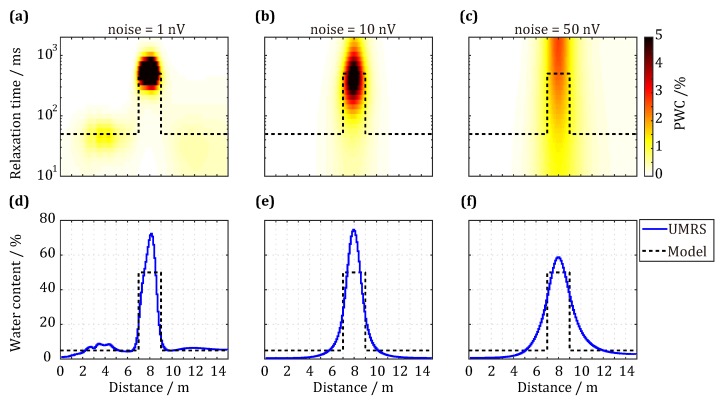
Inversion results of a single water-bearing layer model with a center location of 8 m in front, a water content of 50% and a relaxation time $T_{2}^{*}$ of 500 ms. (**a**–**c**) The distribution of inverted partial water content (PWCs); and (**d**–**f**) the total water content distribution for the noise level 1 nV, 10 nV and 50 nV, respectively.

To test the applicability of the complex QT inversion method for inverting UMRS data, we developed a single water body simulation model. The UMRS data were calculated using the same transmitter/receiver configuration in Figure 2 and 16 pulse moments of 0.01–1 As in the exponential distribution. We added random noise in Gaussian distribution with a standard deviation of 1 nV, 10 nV, and 50 nV to the UMRS data in time domain. The inversion results are shown in Figure 3. The water body is located in front of the detection coil by 7 m–9 m and has a water content of 50% and a relaxation time $T_{2}^{*}$ of 500 ms. When the environment has a relatively low level of noise (1 nV), the distribution of inverted partial water content (PWCs, Figure 3a) is generally consistent with the simulated distribution of our model. The inverting data also show that high water content can be observed for water body locations of 7 m–9 m and $T_{2}^{*}$ values of 350 ms–650 ms. In addition, the inversion results also reflect low-water-content aquifers on the two sides of the water body. Regarding the total water content distribution (Figure 3d), the maximum is 72.35% for a water body location of 8.1 m, and the inverted maximum is higher than the model estimate. When the noise level increases to 10 nV (Figure 3b), the distribution of PWCs is similar to the corresponding simulated distribution, but the inverted $T_{2}^{*}$ values have a wider distribution, and the inversion cannot reflect the low-water-content aquifers. When the noise level continues to increase to 50 nV (Figure 3c), the distribution of PWCs shows significant changes, which means that, in this case, the inversion method can infer only the location and thickness of the water body according to high water content and cannot accurately obtain the distribution of $T_{2}^{*}$ values. Regarding the total water content distribution (Figure 3f), the maximum is 58.67% for a water body location of 8.0 m, which reveals that the QT inversion method can still obtain the distribution of the water body as well as its water content in quite noisy environments. With an increasing level of environmental noise, the inversion accuracy, however, gradually decreases, and, to obtain reliable inversion results, noises clearly need to be maximally reduced.

## 3. Mine Antennae Design

### 3.1. Antenna Structure

**Figure 4:**
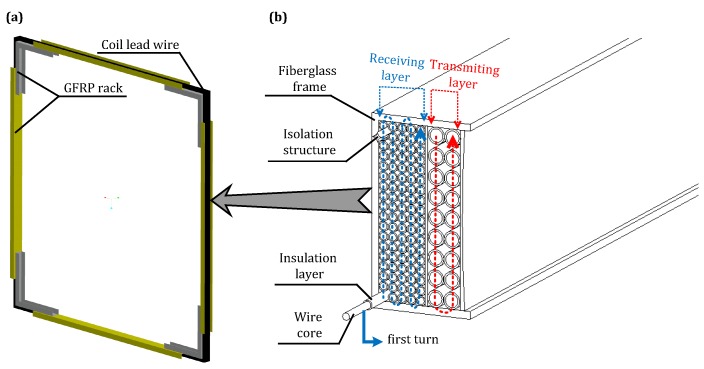
Physical structure diagram of mine meter-scale antenna. (**a**) 2-m stationary detection antenna and bracket; (**b**) antenna internal structure.

The antenna used in this study for UMRS detection in mines is only 2 m in size with an effective surface of 4 m${}^{2}$ for each single turn of the antenna. To improve the amplitude of the UMRS signal, the detection antenna must adopt a multi-turn module. In previous studies, the adopted antennae were usually made of soft and randomly wound coils, and, during different runs of measurements, the electronic parameters were likely to change, thus reducing the detection stability for long-term systematic studies. In this study, our designed antenna was fixed in a special stand to ensure consistent electronic properties. Figure 4a illustrates the integrated design for the 2-m stationary detection antenna, in which the antenna stand is made of U-shaped non-conductive fiberglass material with a divided structure.

Figure 4b represents the physical structure and the interior of the antenna, which shows that the coil system is composed of a transmitting coil with 18 turns for the transmitter in the outer layer and a receiving coil with 90 turns in the inner layer. The receiver and transmitter are separated by a layer of isolation material. The advantage of the separation mode compared to the coincident mode is that the receiver and transmitter can be made using coils of different wire diameters and turns. Since the transmitting coil carries a strong current, it is necessary to use a very thick cable and insulating layer to prevent high-voltage breakdown. The use of large number of turns not only increases the volume and weight, but also increases the inductance and internal resistance, resulting in reduced transmit waveform quality and increased transmitter power requirements. In contrast, the receiving coil can use a small wire diameter cable and large number of turns to obtain higher receiver sensitivity. Each turn of the receiving coil is made of pure annealed multi-core copper material, and pores in between the antenna turns are bonded to the isolation material and silicon rubber to ensure the high-voltage insulation property of the antenna system. More details of the optimal combination of the transmitting and receiving coil can be found in [28].

The antenna system is fixed inside of a fiberglass stand. An antenna can be wound in two different ways, corresponding to regular winding and irregular random winding. Irregularly and randomly wound antennae usually have low distributed capacitances, which can help reach high frequencies but requires high insulation performances of the antennae. Because of the transmit-receive coupling, randomly winding coils must ensure a very high insulation between turns; otherwise, high induction voltage will penetrate the air, causing a short circuit. In this study, we adopted the regular winding method and divided 90 turns of the antenna into five layers with 18 turns lying in each layer. The allowed maximal induced voltage of the antenna corresponds to the induced voltage produced by the distances between the 36 turns, and this insulation requirement can be easily met by using a 3-mm common high-voltage silicon rubber to avoid the turn-to-turn electric breakdown. Meanwhile, according to the test, our regularly wound antenna has a capacitance of 11.5 nF and a cutoff frequency at 9.39 kHz that far exceeds the frequency band of UMRS signals and satisfies the detection requirement.

### 3.2. Matching Circuit

**Figure 5:**
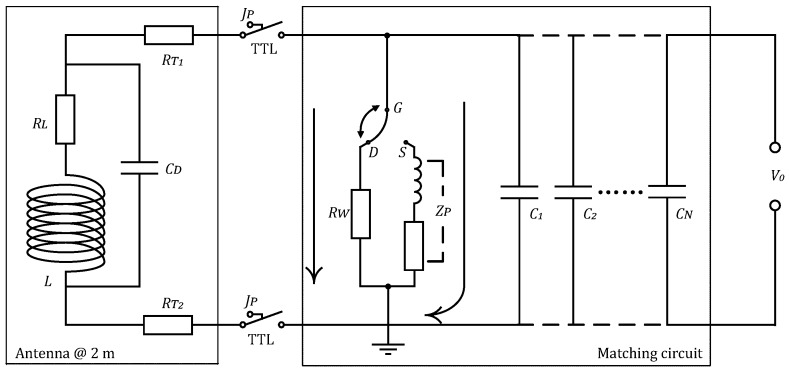
Matching circuit for the multi-turn high inductance antenna.

Figure 5 illustrates the integrated structure of the matching circuit for the receiving antenna, which is mainly composed of an antenna equivalent circuit, a Q-switch circuit, and a frequency selective circuit. The receiving antenna can be expressed by inductance *L*, internal resistance $R_{L}$, and distribution capacitance $C_{d}$. $R_{T1}$ and $R_{T2}$ are contact resistances at antenna interfaces. Since UMRS signals have known frequencies that are related only to the geomagnetic field of the measurement region, we therefore added capacitances, $C_{1}$–$C_{n}$ in the matching circuit, which, together with the antenna inductance *L*, constitute an RLC series-resonant circuit for frequency selection.

**Figure 6:**
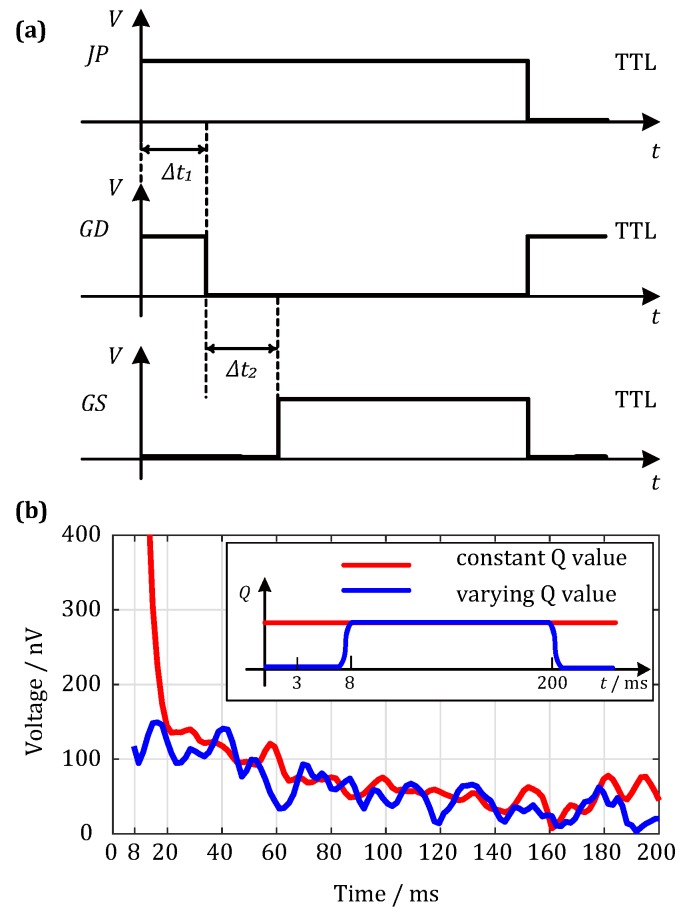
Working flowchart of the Q-switch circuit and the effect to the received signal. (**a**) The operation of the diverter switch G−DS; (**b**) the received signal with a constant Q (red lines) and a varying Q (blue lines).

Jp between the antenna and matching circuits is a high-voltage reed relay for isolating the strong induced voltage produced in the receiving antenna when the transmitting system is at work, which is controlled by a Transistor-Transistor Logic (TTL) signal and can be used to avoid high-voltage-induced damage to the receiving system. Because the high-voltage relay is a mechanical switch, its switching motion will produce jitter, which forms a ringing phenomenon after the high-Q-value receiving antenna and the amplify circuit. Thus, we added a time-varying Q-switch circuit that consists of a non-inductive resistance $R_{W}$, an antenna matching network $Z_{P}$, and a fast-diverter switch *G*-DS. Figure 6a shows a working flowchart of the diverter switch *G*-DS. According to Figure 6a, when the controlling TTL signal for the relay Jp is at a high level, the relay starts to close. During a closing period of $\Delta t_{1}$, the relay is in a mechanical jittering state, and, meanwhile, the fast-diverter switch *G*-DS is at the *G*-*D* end. $R_{W}$ is a precision resistance of extremely small value. Under the effect of this precision resistance, the whole antenna system is at a low Q-value state and does not send the mechanical jittering signals to the detection system. In this way, the effect of the mechanical jittering on the post-stage amplified circuit is suppressed. After a time of $\Delta t_{2}$, the suppression on the mechanism jittering is complete, and the switch is changed to the *G*-*S* end. At this time, the antenna impedance network becomes involved in the antenna system $Z_{P}$, and the antenna switches to a high Q-value state. The UMRS signals acquired by the receiving antenna are amplified based on frequency selection and enter the amplifier detection unit of the equipment system. Figure 6b compares the signals with and without Q-switch. The red line shows that the antenna matching system is always at a high Q-value state, and the ringing that is produced by the high-voltage relay Jp severely affects the waveform of the acquired signals at an early time. In comparison, the blue line represents the acquired data after adopting the Q-varying technique, and its comparison with the red line clearly demonstrates that the mechanical jittering caused by the switching motions of the relay is effectively suppressed. According to experimental adjustment, we found that, with a $\Delta t_{1}$ value of 3 ms and a $\Delta t_{2}$ value of 5 ms, the matching system exhibits its best performance. Therefore, the system’s dead time from hardware is 8 ms.

## 4. Signal Detection System

### 4.1. Hardware Filter Circuit

**Figure 7:**
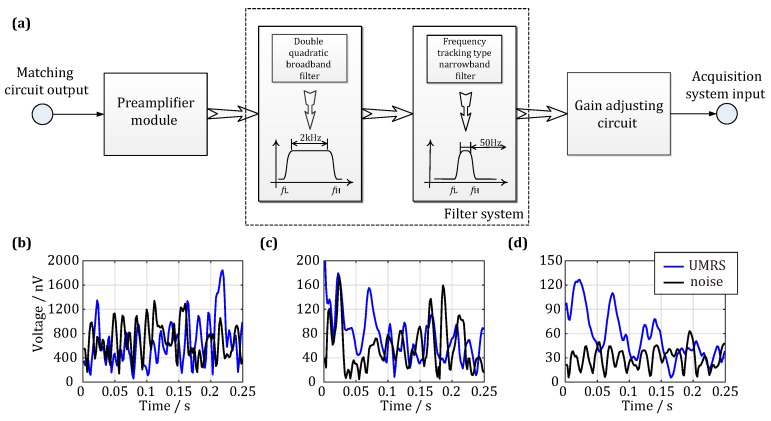
Hardware filter circuit of the UMRS signal detection system. (**a**) Diagram of the filter circuit; (**b**) UMRS signal before passing filter circuit; (**c**) UMRS signal after passing through wideband filter; (**d**) UMRS signal after passing through narrowband filter.

During underground engineering construction, there are usually many pieces of ventilation and lighting equipment, excavation and communication equipment, which are usually densely distributed in the narrow spaces. In turn, the working performance of the UMRS system is severely affected by the electromagnetic interference produced by these pieces of equipment, which must be suppressed by adopting active filtering methods to obtain reliable UMRS signals. The filter system adopted in the UMRS detection system developed in this study is illustrated in Figure 7a. After passing the matching network, the noise-containing signals output by the antenna system are sent to the filter system via a preamplifier that adopts three parallel low-noise amplifier chips as the core amplifier unit [15]. According to tests, the input short-circuit noise level for the preamplifier can reach 1.0 nV$/\sqrt{\mathrm{Hz}}$, and this value satisfies the requirement for detecting weak UMRS signals. The filter system is composed of a broadband filter and a narrowband filter, whose signals are both sent to an analog-to-digital converter (ADC) through a gain-adjustable amplifier for signal conversion and are finally collected by a digital circuit. The broadband filter is a biquadratic filter and constitutes the first level of the filter system with a bandwidth of 2 kHz, a low-frequency cutoff at 1.3 kHz, and a high-frequency cutoff at 3.3 kHz. The frequency range of this filter therefore matches that of UMRS signals. In addition, its pass-band and stop-band gains are 40 dB and −60 dB, respectively, the primary functions of which are to filter out communication noises of very high frequency, power line noise of very low frequency, and spiky noises. The narrowband filter is a frequency-tracking switching capacitor filter with a bandwidth of 50 Hz. Although the UMRS signal with short relaxation times can be distorted such a narrow bandwidth [23,29], the design of this paper is focusing on the water-bearing structures with long relaxation times, which more likely causes water inrush. Its central frequency point can be adjusted according to the frequencies of acquired data. In addition, its pass-band and stop-band gains are 60 dB and −60 dB, respectively, whose primary functions are to filter out power-line harmonic noises and other harmonic noises that are close to the frequencies of UMRS signals. The gain-programmed amplifier is mainly used to dynamically adjust the output signals of the filter according to the digital collection module and practical demands with a dynamic adjustment range of 0 dB–40 dB for the gains.

Figure 7 compares filtered and unfiltered results for a set of UMRS signals. The blue curves in the figure correspond to in situ measured noise-containing signals, and the black curves correspond to the environmental noises only. Both sets of data are gain-normalized. Figure 7b represents the measurement results prior to the filter system and illustrates that UMRS signals are covered by environmental noises with a noise level of 736 nV. Figure 7c represents the same measurement results after the broadband filter but before the narrowband filter, which illustrates that a large portion of the environmental noise is filtered out and the noise level is reduced to 69 nV. At this time, the shape of UMRS signals can be generally recognized based on the filtered data (blue line). Finally, Figure 7d represents the output results after both the narrowband filter and the gain adjustment circuit, which shows that the narrowband filter further suppresses environmental noises that have frequencies away from the bandwidth of UMRS signals; the noise level is further reduced to 32 nV. In addition, the output signal waveform is clearly characteristic of UMRS signals, and the mean square root of the signals is 63 nV. As a result, the SNR of the in situ measurement data increases from −21.4 dB to 5.9 dB. Thus, our in situ measurement results show that the broad- and narrow-band mixed filter system can be used to reliably extract UMRS signals in environments with <−20 dB noises.

### 4.2. Software Noise-Cancelling Scheme

After passing the hardware filter circuit and the ADC in the detection system, the acquired signals still contain a large amount of environmental noise including spiky noises, harmonic noises, and random noises. The spiky noise is a short-time impulse signal of large magnitude, which starts to rapidly decay via oscillation after passing the hardware narrowband filter. Because of its large amplitude and its wide frequency band, this type of noise can lead to large offsets in the obtained UMRS signals. The harmonic noise is mainly caused by high-order harmonics of power line (50 Hz). Although the hardware filter bandwidth is 50 Hz, there are still significant power-line harmonics in a transition zone, corresponding to noises at multiple harmonic frequency points in the filtered results. Finally, the random noise has a reduced waveband after passing the hardware filter, but residual noises still affect the accuracy of the subsequently extracted UMRS signal parameters.

**Figure 8:**
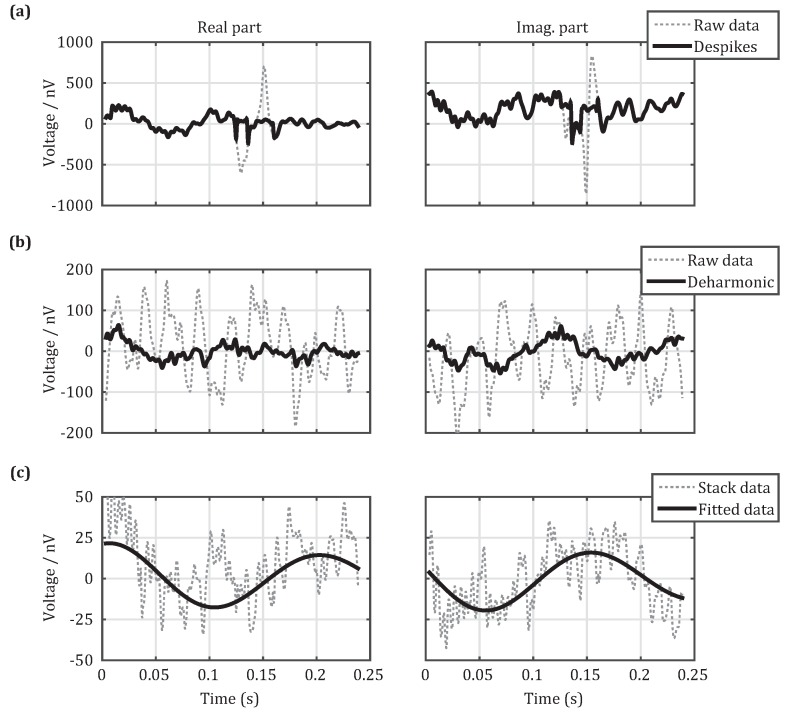
Data processing flow of measured UMRS signal. (**a**) Real and imaginary parts of the measured UMRS signal before and after eliminating spiky noises; (**b**) real and imaginary parts of the UMRS signal after cancelling power-line harmonics; (**c**) real and imaginary parts of the stacked UMRS signal and the fitted results.

To cancel and reduce these three types of noises, we propose the following cancellation schemes, as shown in Figure 8. The first step is to eliminate spiky noises, as shown in Figure 8a. The UMRS signals collected by the detection system are noise-containing data consisting of real and imaginary parts. When spiky noises exist (dotted line), they can be recognized by nonlinear energy operators [17] and statistical criteria [18] and can be replaced by the stacking data from other measurements (solid line). The second step is to cancel out harmonic noises, as shown in Figure 8b. When harmonic noises exist (dotted line), the method of harmonic modeling [16] can be adopted to first search for the base frequencies of power frequency noises that can be then eliminated based on modeling of the acquired data (solid line). The third step is to reduce random noises (dotted line), as shown in Figure 8c. Although random noises cannot be completely eliminated, they can be reduced by stacking the multiple-time measurement data (dotted line). After these three steps, a nonlinear fit method [30] is used to extract the initial amplitude, relaxation time, frequency offset, and initial phase of the UMRS signals. For software data processing, the average SNR reaches above 10 dB and can therefore be used to invert hydrological information on, e.g., water content.

## 5. Field Case of UMRS Detection

### 5.1. Environment of the Study Area

**Figure 9:**
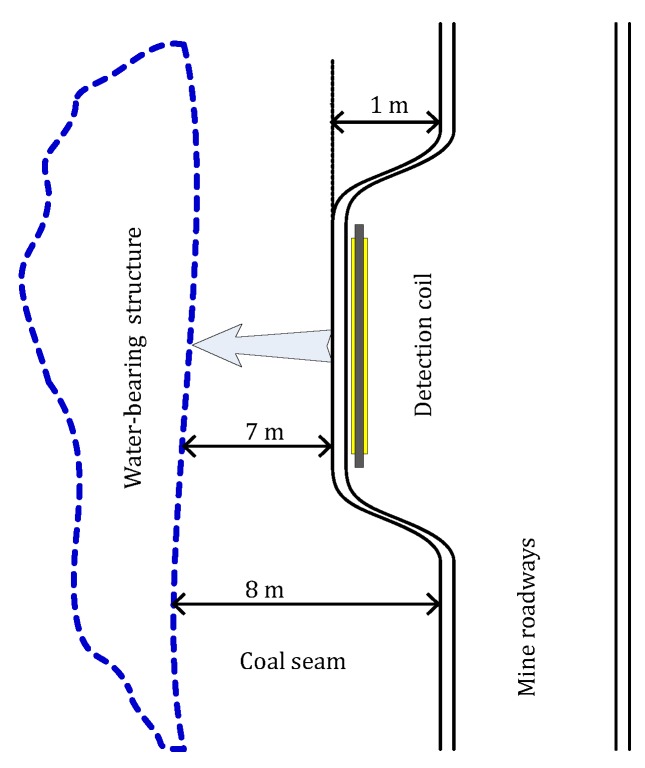
Schematic diagram of measurement site and known water-bearing structure.

The study area is located on a main transport roadway in an active mine in Shanyin County of Shanxi Province, China. The mine lies 350 m below the ground surface, and the geomagnetic field at the test location is 54,650 nT with a corresponding Larmor frequency of 2327 Hz and a geomagnetic inclination of 56.48${}^{\circ }$. In the sidewalls 8 m in front of the test point, there is a large water-bearing structure that formed as a result of collapse and excavation. According to drilling measurements, the water-bearing structure is approximately 1.8 m thick on average, as shown in Figure 9. On one side of the roadway, there is a safe chamber and shelter in the sidewall that is inwardly dished by approximately 1 m, where we set up the 2-m-long stationary square antenna and our designed system. During the detection process, the equipment system conducted five pulse moments of 0.39–1.26 As due to the limited time, and 48 superposed measurements to suppress noises for each pulse moment. The duration of pulse is 40 ms, and the length of data acquisition is 250 ms using a sampling rate of 10 kHz. The dead time of the equipment system was 8 ms, and the filter bandwidth was 56 Hz. Subsequently, the effective dead time including relaxation in the pulse duration [31], system and data processing [32] is –48 ms. The steel reinforcement often used to stabilize the roadways in mines could affect the UMRS measurements. However, in our experiment site, there are some vertical metal anchors above the roof, whose influence on the measurement is negligible.

### 5.2. Results

**Figure 10:**
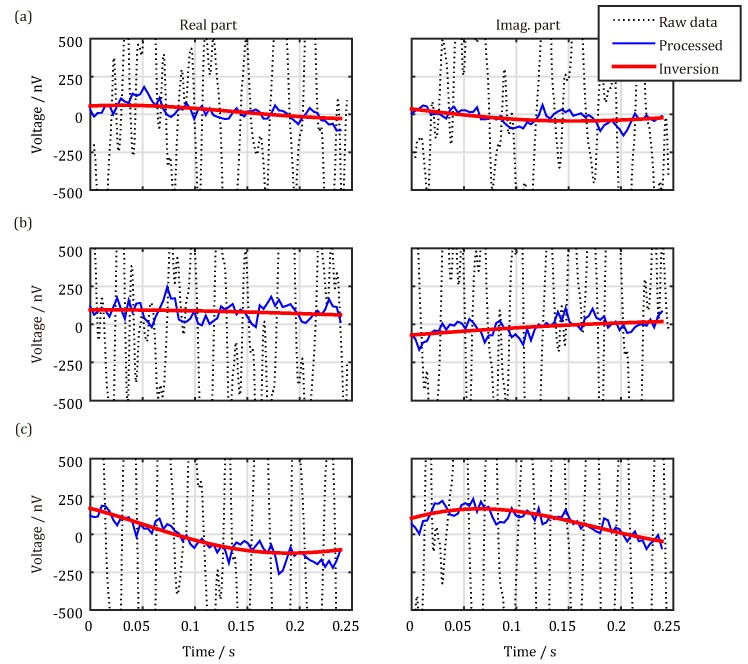
Data processing results of the field measured UMRS signal. The black dashed lines are the raw data, the blue lines are the processed data, and the red ones are the forward-calculated signals from the inversion result model. (**a**) *q* = 0.59 As; (**b**) *q* = 0.81 As; (**c**) *q* = 1.03 As.

Three groups of measurement data are summarized in Figure 10, and the corresponding pulse moments are 0.59, 0.81, and 1.03 As. After removing spiky noises and power-line harmonic noises by software data processing, the SNR is significantly enhanced, as illustrated by Table 1 and the blue solid line in Figure 10. Parameters subsequently extracted from the processed data are shown in Table 1 and are represented by the red solid line in Figure 10. The initial amplitudes of the UMRS signals lie between 63.5 nV and 210.9 nV, and the relaxation times are between 139.7 ms and 497.7 ms; the frequency offsets are lower than 2 Hz, and the average SNR is 14.8 dB, thus satisfying the precision requirement for parameter extraction. Thus, we have demonstrated that our designed meter-scale antenna and the UMRS signal detection system can be used to reliably detect UMRS signals in quite noisy mine environments.

**Figure 11:**
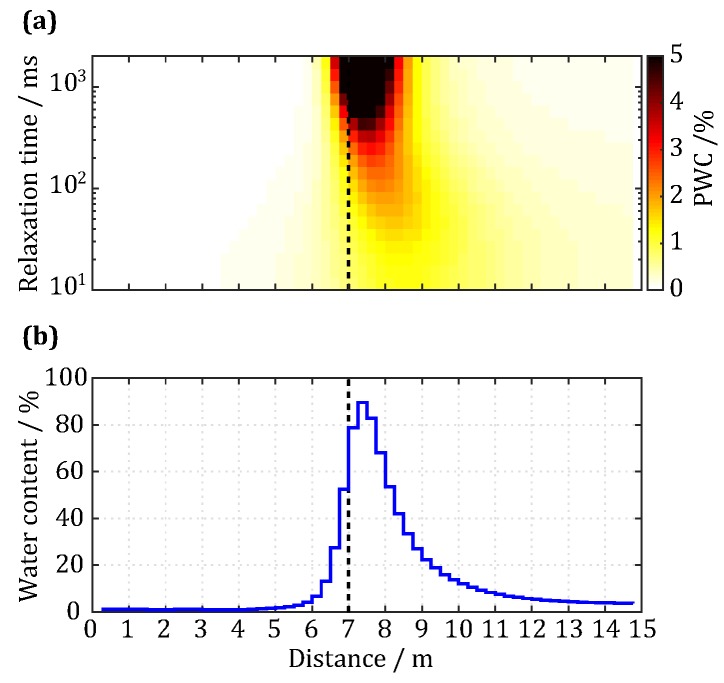
Inversion result of the field measured UMRS signal. (**a**) The distribution of inverted partial water content (PWC); (**b**) the total water content distribution.

Finally, we inverted the processed data by adopting the complex QT inversion method [23] and obtained the distributions of water content and relaxation times for a water body 7–9 m in front of the detection point on the roadway of the mine, as shown in Figure 11. Figure 11a corresponds to the distribution diagram for the PWCs in the front roadway sidewalls, while Figure 11b represents the corresponding distribution diagram for the total water content. For distances smaller than 6 m, the total water content is quite low (<10%); between 7 m and 9 m, the water content rapidly increases to 80%, a water-bearing body can be clearly observed, and the relaxation times mainly lie between 200 ms and 2000 ms; above 8 m, the water content gradually decreases, which is due to two reasons: (1) the water-bearing body is a three-dimensional structure that affects the inversion result in one dimension; and (2) the limit of the number and maximum of pulse moments make the resolution lower at far distances. Although the measurement results of this study were obtained in an environment with a high level of noise (the mean is 52.14 nV), the simulation results in Section 2.2 showed that, at this level of noise, the inversion result could still reflect the location, range, and water content of the water body. Our estimated relaxation times have a fairly wide range, which potentially implies that pores in the water-bearing structure are quite large in size. However, it may also be caused by high noise levels (same situation in Figure 3c) that require more data or other information to verify and validate. This is an open question worth further study. Finally, the known geological data (Figure 9) clearly demonstrate that the water content distribution curve obtained in this study for a water-bearing body 7 m ahead of the measurement point in the roadway walls is consistent with the in situ observation, which thus verifies the effectiveness of the meter-scale antenna and signal detection system proposed in this study.

## 6. Conclusions

UMRS is a novel geophysical method and can be used for detecting water bodies in mines, which could potentially induce geological hazards. However, the problems of limited mine space, complicated environments, and electromagnetic interference severely limit the applicability of this method. In this study, we designed a 2-m-side stationary and multi-turn antenna as well as associated matching circuit, Q-switch circuit, and signal detection and collection systems for the stationary antenna, and we also proposed a software data processing scheme that can be used for reliable extraction of UMRS signals obtained in mines. Finally, we demonstrated the effectiveness of UMRS based on a case study.

Given the narrow spaces of mines, we proposed the use of a 2-m multi-turn detection antenna and a matching circuit for the high-sensitivity antenna, which, compared with an irregular soft antenna, could ensure the stability and consistency of electronic parameters and reduce high-voltage insulation requirements. Meanwhile, the utilization of a Q-switch circuit reduced the transmitting energy and the subsequent effect of mechanical jittering caused by the relay, and thus shortened the dead time to 8 ms.

To solve the problem of severe electromagnetic interference, we adopted an integrated method consisting of a hardware filter circuit and software data processing to lower the noise level and thus improve the SNR. Specifically, we designed a preamplifier and a multi-stage filter module composed of a broadband filter and a narrowband filter to extract reliable signals in a −20 dB noise environment. Subsequently, the filtered data were further processed using our proposed software data processing scheme to eliminate spiky noises and harmonic noises and to reduce random noises, which further improved the SNRs to above 10 dB.

Finally, we applied the meter-scale antenna and the signal detection system developed in this study to conduct a UMRS experiment in a mine of Shanxi, China. Our experimental results showed that this signal detection system could reliably extract UMRS signals with long relaxation times in quite noisy environments. The distribution curves for the water content and the relaxation times that were obtained by inversion of the UMRS signals were generally consistent with the known information on water bodies 7–9 in front of this mine wall, thus demonstrating the effectiveness of the UMRS method and system proposed in this study.

## Figures and Tables

**Table 1:** Fitting parameters of the measured UMRS signals.

Pulse Moment/As	0.39	0.59	0.81	1.03	1.26
initial amplitude/nV	66.7 ± 12.1	63.5 ± 11.4	106.3 ± 13.9	210.9 ± 28.5	144.8 ± 18.9
relaxation time/ms	309.6 ± 15.1	464.3 ± 32.3	497.7 ± 50.5	139.7 ± 19.2	343.0 ± 9.9
frequency/Hz	1.6 ± 0.3	1.6 ± 0.2	1.7 ± 0.2	1.8 ± 0.3	1.6 ± 0.3
phase/rad	33.8 ± 10.3	37.2 ± 10.3	36.1 ± 7.4	37.4 ± 6.9	32.3 ± 10.9
noise/nV	41.7	41.5	52.7	77.4	47.4
SNR/dB	9.4	8.5	14.0	20.0	22.3
